# Assessing the clinical practice in specialized outpatient clinics for chronic obstructive pulmonary disease: Analysis of the EPOCONSUL clinical audit

**DOI:** 10.1371/journal.pone.0211732

**Published:** 2019-02-06

**Authors:** Myriam Calle Rubio, Juan José Soler-Cataluña, José Luis López-Campos, Bernardino Alcázar Navarrete, José Miguel Rodríguez González-Moro, Joan B. Soriano, Manuel E. Fuentes Ferrer, Juan Luis Rodriguez Hermosa

**Affiliations:** 1 Pulmonology Department, Instituto de Investigación Sanitaria del Hospital Clínico San Carlos (IdISSC), Hospital Clínico San Carlos, Madrid, Spain; 2 Medical Department, School of Medicine, Universidad Complutense de Madrid, Madrid, Spain; 3 Pulmonology Department, H. de Arnau de Villanova, Valencia, Spain; 4 Respiratory Disease Medical-Surgical Unit. Instituto de Biomedicina de Sevilla (IBiS), Hospital Universitario Virgen del Rocío/Universidad de Sevilla, Sevilla, Spain; 5 CIBER de Enfermedades Respiratorias (CIBERES), Instituto de Salud Carlos III, Madrid, Spain; 6 Pulmonology Department, Hospital de Alta Resolución de Loja, Granada, Spain; 7 Pulmonology Department, H. Universitario Principe de Asturias, Alcalá de Henares, Madrid, Spain; 8 Instituto de Investigación Hospital Universitario de la Princesa (IISP), Universidad Autónoma de Madrid, Madrid, Spain; 9 Preventative Medicine CMU, Hospital Clínico San Carlos, Instituto de Investigación Sanitaria del Hospital Clínico San Carlos (IdISSC), Madrid, Spain; National and Kapodistrian University of Athens, SWITZERLAND

## Abstract

**Background:**

Chronic obstructive pulmonary disease (COPD) is one of the main reasons for healthcare appointments and use of healthcare resources. In recent years, clinics specializing in COPD have been developed to offer improved care and optimization of recourses for patients with high complexity and frequent decompensations. However, little is known about the clinical practice in this clinical model specializing in COPD. The objectives of this study were to assess the prevalence, characteristics of specialized COPD outpatient respiratory clinics and to evaluate clinical practice in this healthcare model.

**Methods:**

EPOCONSUL is a Spanish nationwide, observational, cross-sectional, clinical audit with prospective case recruitment including the clinical records for 4508 COPD cases from outpatient respiratory clinics over a 12-month period (May 2014–May 2015). The study evaluated clinical practice in 2378 cases from 28 hospitals with both general and specialized COPD outpatient respiratory clinics.

**Results:**

Only 28 (47.5%) centers had an outpatient clinic specializing in COPD, which was characterized by longer patient visits and a higher prevalence of written protocols compared to a general clinic. Patients treated in a specialized clinic had greater obstruction severity, a higher degree of dyspnea and also suffered from more comorbidities. The majority of patients at both types of clinic were classified as high risk (81.1% versus 83%, p = 0.384) according to GesEPOC criteria. Clinical control of COPD was more frequent at specialized clinics, with significant differences in non-severe patients (70.5% versus 56.1%, p < 0.001). Testing was done more frequently in specialized clinics, with better adherence to good clinical practice recommendations.

**Conclusion:**

A specialized COPD outpatient clinic is a healthcare model found in few pulmonology departments that treats more severe patients and those with increased comorbidities. The COPD patients treated in a specialized clinic had a better clinical control, as defined by impact and clinical stability. It is a healthcare model to offer improved care with a higher degree of adherence to guidelines.

## Introduction

Often, one of the most important problems in the structure and organization of healthcare is the management of chronicity and patient complexity. According to data from the World Health Organization (WHO), chronic disease is the cause of 60% of deaths worldwide and results in 75% of public health costs. Furthermore, it is estimated that chronic disease will account for 60% of all disease worldwide by 2020 and will be responsible for 73% of deaths around the world [1–3].

In recent years, important changes have taken place in both the design and organization of healthcare services, such as the distribution of resources according to epidemiological criteria, with the aim of offering healthcare based on greater scientific and technological quality with efficiency criteria [4, 5]. New healthcare strategies need to be applied which focus on more continuous and personalized patient care which is also multidisciplinary, proactive and planned out. Identifying the most appropriate level of care to treat patients is essential to reach a degree of efficiency that allows system sustainability. As a result, in recent years, specific programs have been proposed focusing on healthcare for patients with complex chronic disease such as COPD [6, 7].

COPD is considered the paradigm of chronicity as it is an illness with a high prevalence [8] and morbidity and mortality [9], which is associated with an elevated consumption of healthcare resources [10], thus justifying the implementation of specialized clinics in terms of epidemiology and impact.

A specialized clinic is characterized by a team of expert professionals treating highly complex patients (homogeneous case mix) with the aim of offering comprehensive multidisciplinary care focused on the patient and based on beneficial scientific evidence. This formula has been proven to be ideal in terms of efficiency and quality of patient care, both from the scientific and technical and of course organizational and economic perspectives [11–12]. In recent years, outpatient clinics specializing in COPD have been set up in a number of healthcare centers. However, little is known about the clinical practice in this clinical model specializing in COPD.

The EPOCONSUL study is the first national audit to analyze medical care for COPD in pulmonology departments in Spain. This paper uses the EPOCONSUL database in order to determine the prevalence and characteristics of specialized COPD clinics in pulmonology departments in Spain, as well as to analyze the characteristics of the patients and medical care in specialized COPD clinics.

## Materials and methods

The methodology of the EPOCONSUL audit has been extensively described elsewhere [13]. Briefly, the COPD audit promoted by the Spanish Society of Pulmonology and Thoracic Surgery (SEPAR) was designed to evaluate clinical practice as well as clinical and organizational factors related to managing patients with COPD across Spain. It was designed as an observational, cross-sectional study. Recruitment was intermittent over a single year (May 2014–May 2015). Every 2 months, each investigator recorded the clinical records of the first 10 patients identified as being diagnosed with COPD and seen in the outpatient respiratory clinic (general or specializing in COPD). Subsequently, the patients identified were reevaluated to determine if they met the inclusion/exclusion criteria described in S1 Table. The information collected was historical in nature for the clinical data from the last and previous visits, and information about hospital resources was concurrent. 46 variables associated with the hospitals and 153 associated with the patients were collected. Data from this study is included in S1 File and S2 File.

A total of 59 centers participated (33.7% of those potentially eligible) from 16 of the 17 Spanish autonomous communities (excluding La Rioja). The distribution of hospitals in the different regions and participating investigators are included in S1 Appendix. In order to evaluate the clinical practice for COPD patients in Spain in specialized COPD outpatient respiratory clinics versus general outpatient respiratory clinics, cases audited from 28 hospitals with both general and specialized outpatient respiratory clinics available were analyzed.

The centers were classified according to their size (small or large) as measured by: the number of beds per center ≥500, the number of inpatient respiratory beds ≥20, the number of pulmonology staff members ≥5, and the number of annual outpatient respiratory visits ≥10,000. All the criteria needed to be met to be considered large.

The level of risk was defined according to the Spanish National Guidelines for COPD Care (GesEPOC) criteria (post-bronchodilator FEV1%, degree of dyspnea and history of exacerbations) described in S2 Table [14].

COPD control was evaluated based on two components: impact and stability [15, 16]. Impact can be classified as low or high according to patients' clinical features (degree of dyspnea or COPD Assessment Test score) adjusted for the degree of disease severity defined by FEV1 or even by the BODEx. Stability was defined as the absence of exacerbations in the previous six months. These criteria are described in S3 Table.

In order to evaluate the degree of current CPG implementation of the main recommendations in the 2017 Spanish National Guidelines for COPD Care (GesEPOC) [14] and the 2016 Global Initiative for Chronic Obstructive Lung Disease (GOLD) [17] in both general and specialized outpatient respiratory clinics, we established the cut-off point as fulfilling ≥50% of criteria for good clinical practice evaluated in each category (clinical evaluation of the patient, COPD evaluation and therapeutic intervention).

The protocol was approved by the Ethics Committee at the Hospital Clínico San Carlos (Madrid, Spain; internal code 14/030-E). Additionally, in accordance with current research laws in Spain, the ethics committee at each participating hospital evaluated and agreed to the study protocol. The need for informed consent was waived due to the non-interventional nature of the study, the anonymization of data and the need to blindly evaluate the clinical performance. This circumstance was clearly explained in the protocol and the ethical committees approved this procedure. To avoid modifications to the usual clinical practice and preserve the blinding of the clinical performance evaluation, the medical staff responsible for the outpatient respiratory clinic was not informed about the audit. Data was entered remotely at each participating location to a centrally-controlled server.

### Statistical analysis

The statistical analysis was carried out using the IBM SPSS statistical package (IBM Corporation, Armonk, New York, USA), version 23.0. Quantitative variables were expressed as mean and standard deviation (SD), and median and interquartile range were presented for asymmetric continuous variables. Qualitative variables were expressed by absolute and relative frequency (percentage). The chi-square test was used to compare qualitative variables in the specialized COPD and general outpatient respiratory clinic groups. Quantitative variables in the two risk groups were compared using the Student’s t-test for symmetric variables or the nonparametric Mann–Whitney U test for asymmetric variables. All variables that were statistically significant in the univariate model were included in this model. In all tests of the hypothesis, the null hypothesis was rejected with a type I error or an error of less than 0.05.

## Results

### Population

**Fig 1:**
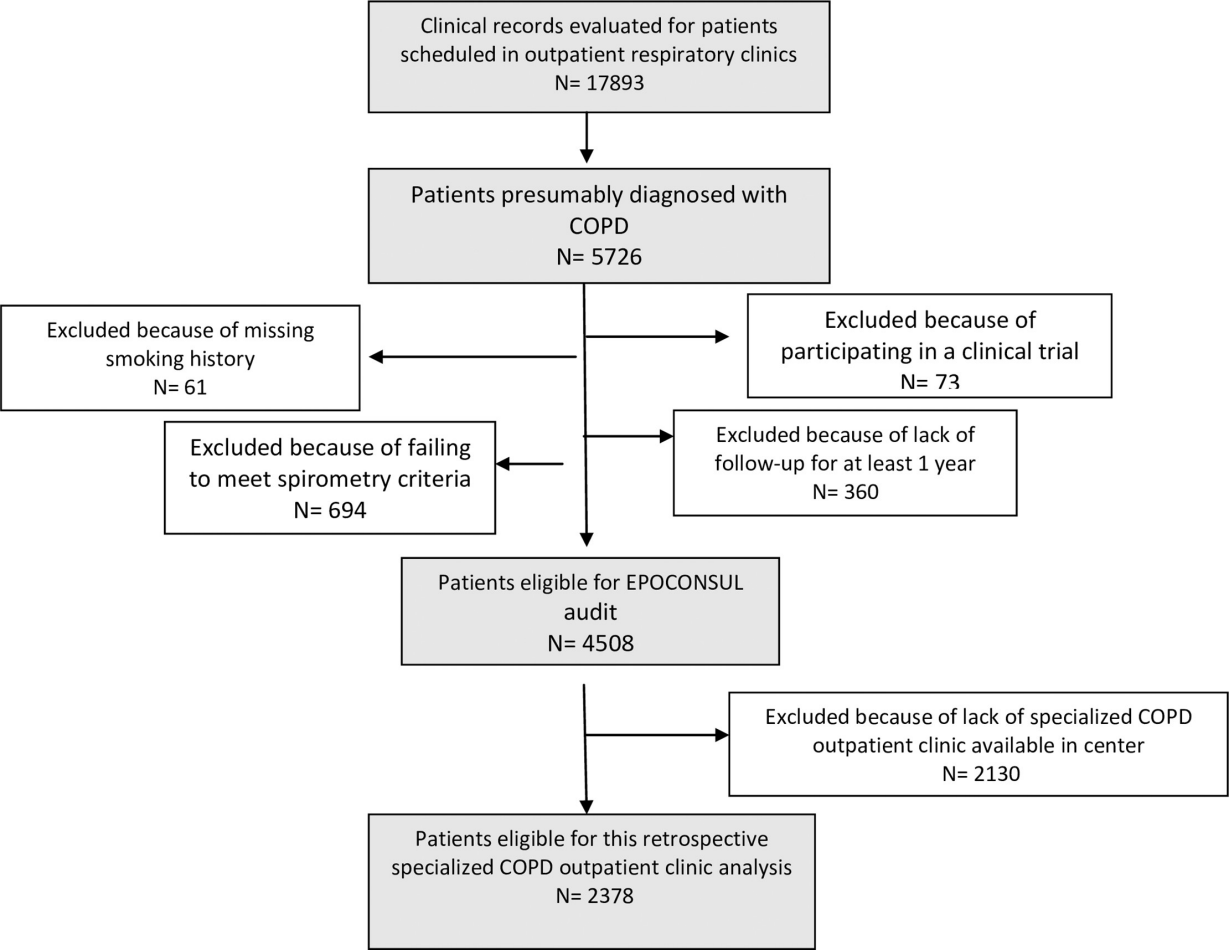
The sampling process described in a STROBE flow chart. A total of 4508 clinical records of patients treated in outpatient respiratory clinics from 59 hospitals were audited. Of this cohort, only 2378 patients audited from 28 hospitals with available outpatient clinic settings (general versus specialized COPD) were included in the analysis.

A total of 4508 clinical records of patients treated in outpatient respiratory clinics from 59 hospitals were audited. Of this cohort, only 2378 patients audited from 28 hospitals with available outpatient clinic settings (general versus specialized) were included in the analysis. The sampling process was detailed in an epidemiology flow chart and described in Fig 1.

### Center characteristics

Hospital center characteristics according to availability of a specialized COPD outpatient clinic are summarized in S4 Table. There were no significant differences found regarding size nor center characteristics such as being a public university center. There were few centers with a specialized COPD outpatient clinic (28 centers, 47.5%).

### Respiratory unit resources

The respiratory unit resources according to availability of a specialized COPD outpatient clinic are summarized in S5 Table. No differences were found in the services portfolio or number of resources available in pulmonology departments according to whether they included a specialized COPD outpatient clinic except for the annual number of visits, which was higher in departments that had a specialized COPD clinic model.

### Outpatient clinic respiratory resources

Available resources according to type of clinic are described in Table 1. In the specialized clinic model, more time was available for the first visit (≥20 minutes, 67.9% versus 50%, p = 0.010) and for follow-up visits (≥15 minutes, 71.4% versus 46.4%, p = 0.039). Protocols were more frequently available in the specialized clinic model (25% versus 3.2%, p = 0.022). No differences were found with regard to nurses available or inhalation therapy educational programs.

**Table 1:** Resources in general and specialized COPD outpatient respiratory clinics.

Characteristics of the outpatient respiratory clinic	General outpatient respiratory clinic	Specialized COPD outpatient clinic	P-Value
**Length of initial outpatient respiratory visit in minutes, median (P25-75)**	19 (15–30)	20 (15–30)	0.063
<20 min, (%)	50	32.1	0.010
≥20 min, (%)	50	67.9	
**Length of follow-up outpatient respiratory visit in minutes, median (P25-75)**	12 (10–15)	15 (12–15)	0.084
<15 min, (%)	53.6	28.6	0.039
≥15 min, (%)	46.4	71.4	
**Nurse available, (%)**	46.4	53.6	0.452
**Inhalation technique educational program available**	25.8	35.7	0.572
**Written COPD protocol available**	3.2	25	0.022

### Audited patient characteristics and clinical conditions

**Fig 2:**
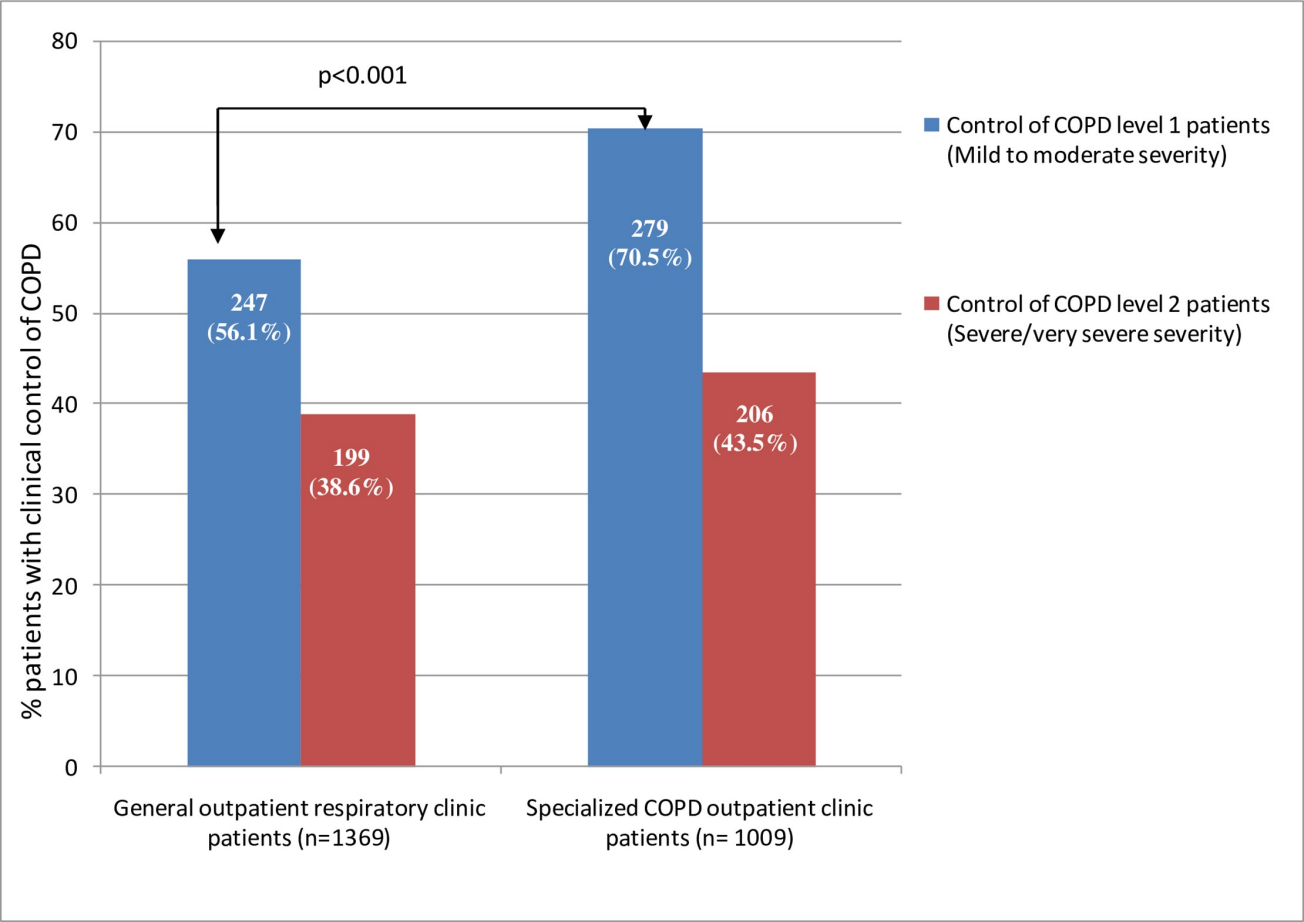
Control of COPD adjusted for severity in both outpatient clinic models. In patients with mild to moderate severity (BODEx ≤2 points or FEV1 ≥50%), to establish a situation of optimal control of COPD, the following criteria must be met: low impact [dyspnea (mMRC) 0–1 and/or CAT ≤10 points] and stability defined as the absence of exacerbations in the last 6 months. In severe/very severe patients (BODEx >2 points or FEV1 <50%), to establish a situation of optimal control of COPD, the following criteria must be met: low impact [dyspnea (mMRC) 0–2 and/or CAT ≤16 points] and stability defined as the absence of exacerbations in the last 6 months.

The demographic and clinical characteristics according to type of clinic are shown in Table 2. Statistically significant differences were found for all variables except sex, age and BMI. Patients treated at a specialized clinic were characterized as having a higher degree of comorbidity according to the Charlson index, and also had a greater degree of dyspnea and more severe degree of obstruction. In addition, other clinical characteristics were more frequent such as the presence of criteria for chronic bronchitis, a symptom suggesting asthma and the emphysema phenotype according to GesEPOC. Triple therapy was the option most commonly used in both types of clinic, although it was more frequent in specialized clinics along with oxygen therapy, home respiratory support and rehabilitation programs. The majority of patients at both the specialized and general clinics were classified as high risk (81.1% versus 83%, p = 0.384). Table 3 shows the distribution of criteria that define high-risk patients according to GesEPOC. Patients meeting the three criteria were more common at specialized clinics (31.9% versus 24.1%, p = 0.014). The most frequent criteria for high risk was a degree of dyspnea ≥2 mMRC at a general clinic and severe obstruction (FEV1 <50%) at specialized clinics. Fig 2 shows the level of COPD control at both types of clinic. Clinical control of COPD was more common in specialized clinics among non-severe patients (70.5% versus 56.1%, p <0.001). There were no significant differences in severe patients (38.6% versus 43.5%, p = 0.159).

**Table 2:** Characteristics of the audited cases at both general and specialized outpatient respiratory clinics.

	Global n = 2378	Global % or median (P25-75)	General outpatient respiratory clinic n = 1369 (57.6%)	Specialized COPD outpatient clinic n = 1009 (42.4%)	P-Value
**Gender (male), (%)**	2378	84.6	84.7	84.5	0.954
**Age (years), m (SD)**	2378	69.37 (9.76)	69.61 (9.88)	69.05 (9.58)	0.164
≤55, (%)		8.5	8.7	8.1	0.199
≥70, (%)		50.5	51.9	48.8	0.342
**Active smokers, (%)**	2378	31.7	38.5	22.4	<0.001
**Smoking pack-years, median (P25-75)**	2378	45(35–68.4)	45 (30–60)	50 (38–79.7)	<0.001
**BMI kg/m2, m (SD)**	2378	27.91 (5.50)	27.86 (5.48)	27.98 (5.53)	0.598
**BMI ≤21 kg/m2, (%)**	2378	8	8.4	7.4	0.401
**Charlson index, m (SD)**	2378	2.82 (2.17)	2.77 (2.18)	2.88 (2.15)	
**Charlson index ≥3, (%)**	2378	43.1	41.3	45.6	0.040
**Dyspnea (MRC-m)**	2378				<0.001
0–1, (%)		27.9	22.9	34.8	
≥2, (%)		45	39.6	52.2	
Missing, (%)		11.3	15.6	5.5	
Level of dyspnea not referred to, (%)		15.8	21.9	7.5	
**CAT questionnaire >10, (%)**	539	64.4	63.7	64.9	0.785
**Chronic bronchitis, (%)**	2378	43	40.1	46.9	<0.001
**Chronic colonization, (%)**	2378	5.3	4.8	6	0.197
**Emphysema phenotype (GesEPOC 2017), (%)**	1145	17.9	15.6	20.5	0.037
**History of asthma or symptoms suggestive of asthma, (%)**	2378	31.5	29	35	0.002
**Post-BD FEV**_**1**_**%, m (SD)**	2378	49.59(17.26)	50.91 (16.96)	47.79 (17.51)	<0.001
**Post-BD FEV**_**1**_ **<50%**		52.5	48.6	57.7	<0.001
**Number of moderate/severe exacerbations in the last year, median (P25-75)**	1708	(0–2)	1.28 (1.48)	1.28 (1.39)	0.874
**Number of hospitalizations in the last year, median (P25-75)**	1472	0.5 (0–1)	0.56(1.00)	0.65 (1.01)	0.052
**High risk level (GesEPOC 2017), (%)**	1388	82	83	81.1	0.384
**BODEx, m(SD)**	197	3.45 (1.95)	3.01 (1.60)	3.84 (2.15)	0.003
**GOLD group, (%)**	590				<0.001
A		22.5	19.3	24.1	
B		18	29.4	12.7	
C		17.8	19.3	17.1	
D		41.7	32.1	46.2	
**COPD Phenotype (GesEPOC 2017)**	2378				<0.001
Non-exacerbator, (%)		28.3	26.5	30.7	
Exacerbator, (%)		19.8	17.5	23	
Missing, (%)		51.9	56	46.3	
**Drug treatment for COPD, (%)**	2391				0.003
LAMA monotherapy		11.9	12.9	10.4	
LAMA-LABA combination		25.4	25.7	24.8	
LABA+ ICS combination		8.1	8.9	7	
Triple therapy		48.3	46.8	50.5	
Quadruple therapy		6.3	5.6	7.4	
**Long-term oxygen therapy, (%)**	2378	26.6	20.5	31.5	<0.001
**Home ventilation, (%)**	2378	8.9	6.6	12.1	<0.001
**Respiratory rehabilitation, (%)**	2378	10.8	7.5	15.4	<0.001

BMI: body mass index; BODE: body mass index, airflow obstruction, dyspnea, and exercise capacity; BODEx: body mass index, airflow obstruction, dyspnea and severe exacerbations; post-BD FEV1%: post-bronchodilator FEV1 percent predicted; Triple therapy: LABA: long-acting beta-2 agonists + LAMA: long-acting antimuscarinic agents + ICS: inhaled corticosteroids; Quadruple: long-acting muscarinic antagonist/long-acting -2 adrenergic agonist/inhaled corticosteroids/other drug (roflumilast or theophylline or long-term antibiotic); GOLD: Global Initiative for Chronic Obstructive Lung Disease; GesEPOC: Spanish National Guidelines for COPD; CAT: COPD Assessment Test.

**Table 3:** Distribution of high-risk patients according to criteria that define their high-risk level at both clinic models.

No. of high-risk criteria met	Patients with a high level of risk in general outpatient respiratory clinics (n = 551)	Patients with a high level of risk in specialized COPD outpatient clinics (n = 587)	P-Value
**A single criterion, n (%)**	**192 (34.8%)**	**189 (32.2%)**	0.014
Only degree of dyspnea ≥2 (mMRC)	105 (54.7%)	67 (35.4%)	
Only FEV1 <50% predicted	44 (22.9%)	81 (42.9%)	
Only ≥2 exacerbations and/or ≥1 hospitalization	43 (22.4%)	41 (21.7%)	
**Two criteria, n (%)**	**226 (41%)**	**211 (35.9%)**	
Degree of dyspnea ≥2 (mMRC) and FEV1<50%	103 (45.6%)	117 (55.5%)	
Degree of dyspnea ≥2 (mMRC) and [≥2 exacerbations and/or ≥1 hospitalization]	78 (34.5%)	57 (27%)	
FEV1 <50% and [≥2 exacerbations and/or ≥1 hospitalization]	45 (19.9%)	37 (17.5%)	
**Three criteria, n (%)**	**133 (24.1%)**	**187 (31.9%)**	

FEV1: post-bronchodilator FEV1 percent predicted; mMRC: modified Medical Research Council.

### Diagnostic procedures during follow-up

The main diagnostic procedures performed during follow-up are described in Table 4. In patients treated at a specialized clinic, it was more common to perform functional evaluation tests such as lung volume measurement (47.6% versus 38.2%; p <0.001), the diffusing capacity test (50.8% versus 45.9%; p <0.02) and the 6-minute walk test (44.2% versus 20.5%; p <0.001). Thoracic computerized axial tomography (CAT) was also more common at specialized clinics (60.7% versus 53.9%; p <0.001).

**Table 4:** Medical care and diagnostic procedures during follow-up in both outpatient clinic models.

	Global (n = 2378)		General outpatient respiratory clinic (n = 1369)	Specialized COPD outpatient clinic (n = 1009)	P-Value
	N	% or median (P25-75)	% or median (P25-75)	% or median (P25-75)	
**Referred from, n (%)**	1857				<0.001
Primary care		41.7	49.3	31.1	
Emergency room		8.5	9.9	6.5	
Other inpatient department		18.8	19.1	18.3	
Other specialties		31	21.6	44	
**Follow-up frequency, n (%)**	2326				<0.001
Less than 6 months		54.6	53.7	55.9	
6–12 months		30.1	33.6	25.4	
More than 12 months		15.3	12.7	18.7	
**Follow-up time (years), median (P25-75)**	2378	4 (2–7)	4 (2–6)	4 (2–6)	0.002
**Bronchodilator reversibility testing, n (%)**	2378	60.7	61.4	59.7	0.396
**Arterial blood gases measured on any occasion, n (%)**	2378	60.8	58.1	64.3	0.003
**Alfa-1- AT serum level testing available, n (%)**	2378	25.7	22.6	29.9	<0.001
**Lung volumes measured on any occasion, n (%)**	2378	42.2	38.2	47.6	<0.001
**Diffusion capacity measured on any occasion, n (%)**	2378	48	45.9	50.8	0.02
**6-min walk test performed on any occasion, n (%)**	2378	30.6	20.5	44.2	<0.001
**Cardiopulmonary exercise testing performed on any occasion, n (%)**	2378	4.4	2.7	6.6	<0.001
**BODE index calculated on any occasion, n (%)**	2378	19.8	11	31.6	<0.001
**Chest CT scan performed on any occasion, n (%)**	2378	56.8	53.9	60.7	<0.001

Abbreviations: Alpha-1 AT: alpha-1 antitrypsin; BODE: body mass index, airflow obstruction, dyspnea and exercise capacity; BODEx: body mass index, airflow obstruction, dyspnea and severe exacerbations; BD test: bronchodilator test; CT: computed tomography; CAT: COPD assessment test.

### Actions taken at the time of the last follow-up visit

Table 5 includes the main clinical actions taken during the last audited visit. At specialized clinics, it was more common to evaluate degree of dyspnea (92.5% versus 78.1%; p <0.001), take a patient history of exacerbations (78.4% versus 67%; p <0.001), comorbidities (82.4% versus 75.1%; p <0.001) and level of physical activity (63.1% versus 37%; p <0.001). Patient characterization by phenotype according to GesEPOC (53.7% versus 44%; p = 0.001), revision of inhalation technique (41.7% versus 27.8%; p = 0.001) and the use of the CAT questionnaire (31% versus 16.5%; p = 0.001) were also more frequent.

**Table 5:** Actions taken at the time of the last follow-up visit in both outpatient clinic models.

	Global (n = 2378)		General outpatient respiratory clinic (n = 1369)	Specialized COPD outpatient clinic (n = 1009)	P-Value
	N	% or median (IR)	% or median (IR)	% or median (IR)	P
**Evaluation of dyspnea severity, n (%)**	2378	84.2	78.1	92.5	<0.001
**Number of moderate or severe exacerbations in the last 12 months recorded, n (%)**	2378	71.8	67	78.4	<0.001
**Data on regular exercise recorded, n (%)**	2378	48.1	37	63.1	<0.001
**Comorbidities identified in the medical record, n (%)**	2378	78.2	75.1	82.4	<0.001
**COPD severity defined in the report by which criteria, n (%)**	2378	74.4	66.6	85	<0.001
FEV1		80.2	85.1	74.9	
BODE		14.5	7.9	21.6	
BODEx		5.3	7	3.5	
**COPD GOLD type defined in the report, n (%)**	2378	24.8	13.7	39.9	<0.001
**COPD phenotype according to GesEPOC defined in the report, n (%)**	2378	48.1	44	53.7	<0.001
**CAT questionnaire completed on any occasion, n (%)**	2378	22.7	16.5	31	<0.001
**Treatment adherence evaluated, n (%)**	2378	46.4	34.6	62.3	<0.001
**Inhalation technique evaluated, n (%)**	2378	33.7	27.8	41.7	<0.001
**Degree of satisfaction with inhalation device evaluated, n (%)**	2378	21	18.4	24.5	<0.001
**Adverse effects of medication recorded, n (%)**	2378	28.9	22.1	38.2	<0.001
**Specific intervention for smoking cessation in active smokers offered, n (%)**	1625	27.7	22.4	33.4	<0.001
**Regular exercise recommended during the visit, n (%)**	2378	49.8	37	67.2	<0.001
**Influenza annual vaccination recorded, n (%)**	2378	50.2	41	62.6	<0.001
**Pneumococcal vaccination recorded, n (%)**	2378	29.2	27.8	31.1	**0.083**
**Any change in current medication advised, n (%)**	2378	22.5	20.7	24.8	**0.022**

### Adherence to good clinical practice criteria

Table 6 describes the adherence to the main recommendations in clinical practice guidelines (CPG) at both types of clinic, showing that specialized clinics better adhere to the main CPG recommendations.

**Table 6:** Adherence to recommendations (GOLD and GesEPOC) in both outpatient clinic models.

Criteria of good clinical practice evaluated in EPOCONSUL	No. of criteria met	Global (n = 2378), (%)	Patients in general outpatient respiratory clinics (n = 1369), (%)	Patients in Specialized COPD outpatient clinic (n = 1009), (%)	P-Value
**During clinical evaluation** ** 1. Is dyspnea severity evaluated on current visit?** ** 2. Is the number of hospitalizations in the last 12 months recorded during current visit?** ** 3. Is the number of moderate or severe exacerbations in the last 12 months recorded during current visit?** ** 4. Is current smoking habit recorded?** ** 5. Is regular exercise data recorded during current visit?** ** 6. Are comorbidities identified in the clinical record?**	6 criteria	22.3	17.8	27.6	<0.001
	>3 criteria	66.9	57.6	79.5	<0.001
	≤3 criteria	33.1	42.4	20.5	<0.001
**During COPD evaluation** **1. Is alfa-1-antitrypsin serum level determination available?** **2. Is COPD severity defined in the report?** **3. Is COPD GOLD type defined in the report?** **4. Is COPD phenotype according to GesEPOC defined in the report?** **5. Is the 6MWT performed on any occasion?** **6. Is diffusion capacity measured on any occasion?** **7. Are lung volumes measured on any occasion?** **8. Is a chest CT scan performed on any occasion in the exacerbator phenotype?**	8 criteria	2.1	0.6	4.3	<0.001
	>4 criteria	32.6	23.9	44.4	<0.001
	≤4 criteria	67.4	76.1	55.6	<0.001
**During therapeutic intervention** **1. Is treatment adherence evaluated in any way?** **2. Is inhalation technique evaluated in any way?** **3. Is pneumococcal vaccination recorded?** **4. Is exercise advised during the visit?** **5. Have arterial blood gases been measured on any occasion for patients on long-term oxygen therapy?**	5 criteria	11.6	9.1	14.9	<0.001
	>3 criteria	28.1	21.5	37.1	<0.001
	≤3 criteria	71.9	78.5	62.9	<0.001

In order to evaluate the degree of current CPG implementation of the main recommendations, we evaluated the number of criteria for good clinical practice met in each category (clinical evaluation of the patient, COPD evaluation and therapeutic intervention).

## Discussion

This paper provides data on clinical practice and the characteristics of patients treated in specialized COPD outpatient clinics compared to general clinics in pulmonology departments in Spain for the first time.

COPD is a widely prevalent disease, 10% in those aged 40–80 [18]. The disease is progressive and is often associated with a high degree of comorbidity and mortality, ranked as the fifth leading cause of death in Spain at present [19, 20]. It is currently a priority in healthcare system plans [21] as it is associated with a substantial demand for care. COPD is one of the main reasons for healthcare appointments and use of healthcare resources, both for primary and specialized care. In Spain, the disease accounts for 10–12% of primary care visits, 35–40% of pulmonology visits and is responsible for 7% of hospitalizations [22]. The average healthcare cost generated by each Spanish patient is estimated to be 3538 euros/year, of which more than 85% corresponds to costs for hospitalizations due to exacerbations [23]. As a result, patients with highly complex cases and frequent decompensation will have a higher social health impact due to frequent hospitalizations [24].

Systematized healthcare has been shown to improve quality of life and prognosis in complex chronic patients, in addition to lowering the cost of care (reducing hospitalizations and length of hospital stays, lowering the number of ER visits, appropriate use of medication, etc.) [25, 26]. This data warrants the need to adapt current mechanisms in COPD care and to reshape the care model, guaranteeing specialized units for patients with high complexity and frequent decompensations such as specialized clinics. As a result, clinics specializing in COPD have been developed in recent years to offer improved care and optimization of recourses [27, 28, 12].

The results of our study show that a clinic specializing in COPD is a healthcare model found in few pulmonology departments (in 47.5%), despite the fact that the majority of the hospitals participating in the audit were public university hospitals with training for medical specialists. We found no differences with regard to specialized clinic availability, center characteristics or the resources available in the pulmonology department such as medical staff or diagnostic procedures, except for a higher number of visits to pulmonology departments that included a clinic specializing in COPD. The low prevalence of clinics specializing in COPD in Spain is not in line with current guidelines established in COPD action plans for patients with a high degree of intervention and greater complexity [29, 21] in which follow-up falls on the referred hospital specialist in a separate clinic characterized by a higher degree of specialization and training in COPD.

With regard to available resources, our study found that more time was available for both the first visit and follow-up visits at specialized clinics and protocols were also more commonly available. These two tools are considered crucial, since having ample time during the visit improves communication with the patient, reduces the need for tests and reduces possible mistakes in treatment [30]. The application of protocols and clinical guidelines is also a fundamental tool to improve efficiency and reduce variability in care provided [31].

In our study, patients treated at clinics specializing in COPD had a higher rate of comorbidity. Chronic expectoration and the emphysema phenotype were also more common. These findings are in line with numerous studies that have shown that the presence of comorbidities, cough with chronic expectoration and emphysema are associated with a higher risk of exacerbations, a worse prognosis and higher mortality [32, 33]. In both general outpatient respiratory clinics and those specialized in COPD, the majority of COPD patients were classified as high risk according to the GesEPOC criteria (post-bronchodilator FEV1%, degree of dyspnea and history of exacerbations) described in S2 Table [13, 34], although it must be noted that patients who met the three criteria defining high risk with a higher degree of dyspnea and obstruction severity were more common at specialized clinics. These differential aspects in the clinical characteristics of patients and the origin of the visit could suggest that, at centers with both types of clinics, patients who are more complex or “fragile” or the patients who need complex treatment (home ventilation or respiratory rehabilitation) are strategically selected for referral to the specialized COPD outpatient respiratory clinics from other specialties rather than the general outpatient respiratory clinic to which patients are generally referred from primary care. Nevertheless, one limitation to bear in mind is that referral criteria could not be evaluated as it was not available.

A relevant result is the fact that patients treated in a specialized clinic, despite being more severe in terms of disease burden, had a better clinical control of their COPD, as defined by impact and clinical stability [15], with significant differences in patients with a mild or moderate degree of severity (70.5% versus 56.1%, p <0.001). This data suggests that clinical control may be an achievable therapeutic objective for a significant number of patients with COPD in a healthcare model with a higher degree of adherence to guidelines like the specialized clinic, according to information from our study. Nevertheless, this is a cross-sectional analysis that does not evaluate long-term clinical data.

As far as medical care according to type of clinic, it must be noted that there are differences in diagnostic and therapeutic procedures, as it is more common to complete specific tests in a specialized clinic such as measuring lung volume, the diffusing capacity test, computerized axial tomography (CAT), or the 6-minute walk test. Actions to evaluate treatment are also more common, with better adherence to good clinical practice recommendations. These differences in actions performed would not be explained by level of patient risk, and may be related to the use of protocols or clinical pathways, having more time in the clinic and greater knowledge and interest among the professionals treating patients in the specialized clinic model, although this aspect was not evaluated. In this sense, numerous studies have shown a notable variability in COPD care in different environments which is not exclusively influenced by clinical presentation or resources available, but instead is explained by a grouping or clustering effect [35–37] based on the center or care model. This is why management using integrated care processes and care protocols according to principles of evidence-based medicine are fundamental tools to improve efficiency, reduce variability in clinical practice and contribute to improving the quality of care. However, it is important to remember that while CPG recommendations are based on scientific evidence in order to systematize actions, COPD is a heterogeneous and complex disease with a variable clinical presentation [38].

With regard to the type of treatment according to clinic type, we must note that a high percentage of patients received inhaled corticosteroids at both types of clinic, in accordance with other national studies done at different levels of care [39], although the results of our analysis showed that triple therapy, oxygen therapy and home respiratory support were more common in specialized clinics. A change in drug treatment was also more common in specialized clinics, which may reflect more proactive care in these specialized units. However, there was no change in prescription for the majority of patients (75.2%). This infrequent changing of drug treatment in pulmonology departments is consistent with other studies in which it was associated with the presence of clinical characteristics such as a lower frequency of exacerbations and symptoms, which identify better management in a patient [40].

Finally, a few methodological limitations should be kept in mind such as the fact that center selection was not randomized and was based on having previously participated in COPD clinical audits. We must also consider the limitation intrinsic to any clinical audit, the fact that some values were not recorded as they were not available. Additionally, we must mention the possible limitation that this was a cross-sectional study, so differences in risk of exacerbations or complications compared to patients treated in general respiratory clinics cannot be evaluated. Further clinical audits are necessary to evaluate the impact on clinically significant results. However, despite these limitations, we believe that due to its population coverage, the sample included is representative of medical attention for patients with COPD in Spain according to the type of outpatient respiratory clinic.

## Conclusions

A specialized COPD outpatient respiratory clinic is a healthcare model present in very few pulmonology departments in Spain. It is characterized by a greater amount of time reserved for patient care and creating protocols for care, although it does not have additional human resources or equipment available. This formula has been proven to offer a better clinical control of COPD with greater adherence to clinical practice guidelines. However, future studies are needed in order to evaluate whether it has an impact on clinically relevant results.

## Supporting information

S1 Appendix Investigators participating in the EPOCONSUL study. (DOCX)

S1 Table The inclusion criteria and exclusion criteria. (DOCX)

S2 Table Risk stratification according to GesEPOC. (DOCX)

S3 Table COPD management criteria with adjustment for severity according to the BODEx index or FEV_1_%. (DOCX)

S4 Table Hospital center characteristics according to availability of a specialized COPD outpatient clinic. (DOCX)

S5 Table Respiratory unit resources according to availability of a specialized COPD outpatient clinic. (DOCX)

S1 File Data from this study. (XLSX)

S2 File Data from this study. (XLSX)

## Abbreviations

COPD: chronic obstructive pulmonary disease
CPG: clinical practice guidelines
GesEPOC: Spanish National Guidelines for COPD care
IQR: interquartile range
BMI: body mass index
LAMA: long-acting antimuscarinic agents
LABA: long-acting beta-2 agonists
ICS: inhaled corticosteroids
