# Supplementary material for: Assessing the clinical practice in specialized outpatient clinics for chronic obstructive pulmonary disease: Analysis of the EPOCONSUL clinical audit
Source: PLoS One. 2019 Feb 6;14(2):e0211732. doi: 10.1371/journal.pone.0211732 (PMC6364994; doi:10.1371/journal.pone.0211732)
Supplement: S1 Table — (DOCX) [file pone.0211732.s002.docx]

**S1 Table**

Title: Inclusion and exclusion criteria.

| Inclusion criteria | - patients aged ≥40 years  - smokers or ex-smokers (≥10 pack-years)  - COPD diagnosed based on spiro­metry tests (post-bronchodilator FEV1/FVC <0.7 or pre-bronchodilator FEV1/FVC <0.7 and FEV1 ≤80% if no bronchodilation reversibility testing is available) |
| --- | --- |
| Exclusion criteria | - lack of follow-up for at least 1 year in an outpatient respiratory clinic  - participating in a clinical trial |
