# Supplementary material for: Assessing the clinical practice in specialized outpatient clinics for chronic obstructive pulmonary disease: Analysis of the EPOCONSUL clinical audit
Source: PLoS One. 2019 Feb 6;14(2):e0211732. doi: 10.1371/journal.pone.0211732 (PMC6364994; doi:10.1371/journal.pone.0211732)
Supplement: S2 Table — (DOCX) [file pone.0211732.s003.docx]

**S2 Table**

Title: Risk stratification according to GesEPOC.

**Risk level stratification**

**LOW RISK**

**(must meet all criteria)**

**HIGH RISK**

**(must meet at least one criterion)**

**<50%**

**Obstruction**

**(Post-bronchodilator FEV1%)**

**≥50%**

**>2 or =2 (with treatment)**

**Dyspnea (mMRC)**

**0 – 2**

**0 – 1 exacerbations (without hospitalization)**

**≥2 exacerbations or**

**≥1 hospitalization**

**Exacerbations in the last year**

**Appendix S4**
