# Supplementary material for: Assessing the clinical practice in specialized outpatient clinics for chronic obstructive pulmonary disease: Analysis of the EPOCONSUL clinical audit
Source: PLoS One. 2019 Feb 6;14(2):e0211732. doi: 10.1371/journal.pone.0211732 (PMC6364994; doi:10.1371/journal.pone.0211732)
Supplement: S3 Table — (DOCX) [file pone.0211732.s004.docx]

**S3 Table**

Title: COPD management criteria with adjustment for severity according to the BODEx index or FEV_1_%.

| **CLINICAL EVALUATION** | **COPD management criteria with adjustment for the BODEx index or FEV_1_%** | |
| --- | --- | --- |
| **Low clinical impact** (at least 1 of the 2 criteria must be met) | | |
|  | FEV_1_ ≥50%  (or BODEx ≤2) | FEV_1_ ≤49%  (or BODEx >2) |
| Dyspnea | 0-1 | 0-2 |
| CAT | 0-10 | 0-16 |
| **Clinical stability** (criteria must be met) | | |
| Exacerbations in the last 6 months | None | |
| **COPD management** | Low impact + stability | |

Legend: COPD control was evaluated based on two components: impact and stability. Impact can be classified as low or high according to patients' clinical features (degree of dyspnea or COPD Assessment Test score) adjusted for the degree of disease severity defined by FEV1 or even by the BODEx. Stability was defined as the absence of exacerbations in the previous six months.
