# Supplementary material for: Assessing the clinical practice in specialized outpatient clinics for chronic obstructive pulmonary disease: Analysis of the EPOCONSUL clinical audit
Source: PLoS One. 2019 Feb 6;14(2):e0211732. doi: 10.1371/journal.pone.0211732 (PMC6364994; doi:10.1371/journal.pone.0211732)
Supplement: S4 Table — (DOCX) [file pone.0211732.s005.docx]

**S4 Table**

Title: Hospital center characteristics according to availability of a specialized COPD outpatient clinic.

| **Characteristics** | **Center without specialized COPD outpatient clinic** | **Center with specialized COPD outpatient clinic** | **P-Value†** |
| --- | --- | --- | --- |
| Number of participating hospitals, n= 59 | 31 | 28 |  |
| Large hospital, (%) | 48.4 | 60.7 | 0.435 |
| University hospital, (%) | 77.4 | 89.3 | 0.306 |
| Public hospital, (%) | 93.5 | 92.9 | 1 |
| Beds per center ≥500, (%) | 58.1 | 67.9 | 0.591 |
| Beds per center, median (P25-75) | 26 (19-37.5) | 32 (25.7-34.5) | 0.163 |

Legend: The hospital center was considered large if the number of beds per center was ≥500, the number of inpatient respiratory beds ≥20, the number of pulmonology staff members ≥5, and the number of annual outpatient respiratory visits ≥10,000. All the criteria must be met.
