# Supplementary material for: Assessing the clinical practice in specialized outpatient clinics for chronic obstructive pulmonary disease: Analysis of the EPOCONSUL clinical audit
Source: PLoS One. 2019 Feb 6;14(2):e0211732. doi: 10.1371/journal.pone.0211732 (PMC6364994; doi:10.1371/journal.pone.0211732)
Supplement: S5 Table — (DOCX) [file pone.0211732.s006.docx]

**S5 Table**

Title: Respiratory unit resources according to availability of a specialized COPD outpatient clinic.

| **Respiratory unit resources** | **Respiratory unit without specialized COPD outpatient clinic** | **Respiratory unit with specialized COPD outpatient clinic** | **P-Value†** |
| --- | --- | --- | --- |
| **Inpatient respiratory clinic available, (%)** | 80.6 | 85.7 | 0.734 |
| **Number of inpatient respiratory beds ≥20, (%)** | 76 | 91.7 | 0.247 |
| **Number of pulmonology staff members ≥5, (%)** | 80.6 | 82.1 | 1 |
| **Pulmonology residents available, (%)** | 61.3 | 75 | 0.282 |
| **Number of annual outpatient respiratory visits, median (P25-75)** | 12500 (9289-22890) | 17745 (13882-27299) | **0.008** |
| **Number of annual outpatient respiratory visits ≥10,000, (%)** | 71 | 92.9 | **0.045** |
| **Nursing outpatient respiratory clinic available** | 45.2 | 46.4 | 1 |
| **Functional respiratory laboratory available** | 100 | 100 | 1 |
| **6MWT available** | 90.3 | 100 | 0.239 |
| **Inhalation technique educational program available** | 25.8 | 35.7 | 0.572 |
| **Cardiopulmonary exercise testing available** | **51.6** | **75** | 0.105 |
| **Respiratory rehabilitation program available**   \| Hospital-based \|  \| \| --- \| --- \| \| Home-based \|  \| \| Mixed \| \| | 64.5  55  10  35 | 85.7  66.7  4.2  29.2 | 0.078  0.637  0.635  1 |
| **Alfa-1-antitrypsin genetic testing available** | 71 | 64.3 | 0.781 |
| **Sputum eosinophil count available** | 48.4 | 39.3 | 0.601 |
